# Origin and invasion of the emerging infectious pathogen *Sphaerothecum destruens*

**DOI:** 10.1038/emi.2017.64

**Published:** 2017-08-23

**Authors:** Salma Sana, Emilie A Hardouin, Rodolphe E Gozlan, Didem Ercan, Ali Serhan Tarkan, Tiantian Zhang, Demetra Andreou

**Affiliations:** 1 Bournemouth University, Faculty of Science and Technology, Fern Barrow, Talbot Campus, Poole, Dorset BH12 5BB, UK; 2 UMR BOREA IRD-MNHN-Université Pierre et Marie Curie, Muséum National d’Histoire Naturelle, 47 Rue Cuvier, Paris, Cedex 5 75231, France; 3 Faculty of Fisheries, Muğla Sıtkı Koçman University, Kötekli, Muğla 48000, Turkey

**Keywords:** aquaculture, biological invasion, fungal pathogens, invasive

## Abstract

Non-native species are often linked to the introduction of novel pathogens with detrimental effects on native biodiversity. Since *Sphaerothecum destruens* was first discovered as a fish pathogen in the United Kingdom, it has been identified as a potential threat to European fish biodiversity. Despite this parasite’s emergence and associated disease risk, there is still a poor understanding of its origin in Europe. Here, we provide the first evidence to support the hypothesis that *S. destruens* was accidentally introduced to Europe from China along with its reservoir host *Pseudorasbora parva* via the aquaculture trade. This is the first study to confirm the presence of *S. destruens* in China, and it has expanded the confirmed range of *S. destruens* to additional locations in Europe. The demographic analysis of *S. destruens* and its host *P. parva* in their native and invasive range further supported the close association of both species. This research has direct significance and management implications for *S. destruens* in Europe as a non-native parasite.

## INTRODUCTION

Fungal parasites have emerged as serious threats to biodiversity in the past 20 years, resulting in the worldwide decline of several taxa.^1^ Facilitated by the international trade in live animals, diseases such as chytridiomycosis and white-nose syndrome have resulted in high levels of mortalities in amphibian and North American bat populations,^2, 3, 4, 5^ respectively. Emergent fungi are frequently generalists, and they can survive in a wide range of temperatures. They are often transmitted both directly and indirectly, enabling their transport and introduction to native communities across the globe.^1, 6, 7^ Wild population declines are difficult to detect, especially for aquatic species.^8^

Long-term monitoring of wild fish populations in combination with histological checks has provided the first empirical evidence that links the fungus-like parasite *Sphaerothecum destruens* to declines in endemic fish species in Turkey.^9^ This parasite was first identified in Europe in 2005 after it caused high fish mortalities in semi-natural and lab experiments.^10^ Its reservoir host, the invasive fish *Pseudorasbora parva*, is the suspected source of the parasite in Europe. *P. parva* was accidentally introduced to Europe from China and has invaded 32 countries in <40 years.^11^ As it was first reported in Europe, *S. destruens* has been highlighted as a true generalist and an important pathogen of fishes, with over 14 species as its hosts (including important aquaculture species, such as salmon, carp and sea bass).^9, 12, 13^ Currently, there are two known geographically distinct strains of this parasite—one in North America and one in Eurasia.^14^

Disease outbreaks by *S. destruens* have been reported in both wild and cultured salmonid fishes on the west coast of the USA.^15, 16^ It has caused high mortality in cultured Chinook salmon (*Oncorhynchus tshawytscha*)^15, 17^ and in subadult Atlantic salmon (*Salmo salar*).^16^ Further work has confirmed the susceptibility of other salmonids (Coho salmon (*O. kisutch*), rainbow trout (*O. mykiss*) and brown trout (*S. trutta*)).^17^ The European strain was first detected in the cyprinid fishes sunbleak (*Leucaspius delineatus*) and fathead minnow (*Pimephales promelas*) with high mortalities.^10^ Experimental infections with the European strain have confirmed that it can cause the same pathology and morbidity in *S. salar* as the North American strain.^13^ Furthermore, the European strain can cause mortality in more cyprinid fishes, such as carp (*Cyprinus carpio*), roach (*Rutilus rutilus*), bream (*Abramis brama*), rudd (*Squalius fellowesii*)^12^ and topmouth gudgeon (*Pseudorasbora parva*),^18^ as well as *Oxynoemacheilus* sp. (Family Nemacheilidae) and *Lepomis gibbosus* (Family Centrachidae).^9^ Despite the parasite’s emergence and associated disease risk, there is still a poor understanding of its origin in Europe.

*S. destruens* is an obligate intracellular parasite belonging to the Rhinosporideacae family and the Mesomycetozoea class.^19^ Within its fish host, *S. destruens* spores can infect multiple organs (liver, kidney, gonads, intestine, and gills^9^). The spores divide asexually within the cells, eventually leading to cell death, releasing the spores, and these spores then infect new cells. Spores are released in urine and seminal fluids and can cause infection to native fish through both direct transmission (contact with spores) and indirect transmission. Indirect transmission is facilitated by zoospores, which are produced once the spores are incubated in freshwater.^19^ Zoospores and spores have a wide temperature tolerance (5–30 °C), potentially facilitating the spread of this parasite to new environments.^6^ Within tissues, *S. destruens* can cause disseminated and granulomatous histopathology.^20^

In this paper, we provide the first evidence that *S. destruens* has invaded Europe from China along with its reservoir host, the highly invasive small cyprinid species *P. parva.* This result was achieved through extensive sampling of *P. parva* across its native and invasive range, coupled with pathogen detection in these populations. The close links of the reservoir host with aquaculture and the high susceptibility of aquaculture and native species to *S. destruens* raise serious threats to European freshwater biodiversity with important policy implications.

## MATERIALS AND METHODS

### *S. destruens* detection

**Figure 1:**
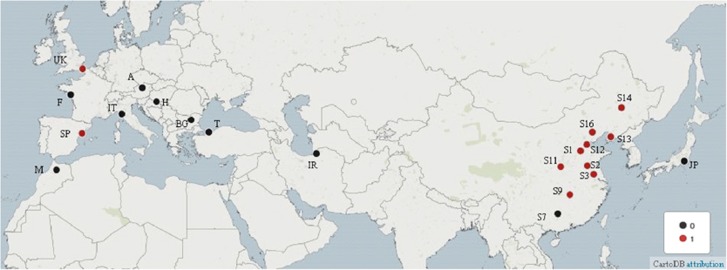
*Pseudorasbora parva* sites screened in this study for *Sphaerothecum destruens* presence in the host’s native range (China) and invasive range (Europe, North Africa and Eurasia). The red sites were positive for *S. destruens* and the black sites were negative using the 18S rRNA marker. Europe- Austria, A; Bulgaria, BG; France, F; Hungary; H, Italy, IT; Spain, SP; Turkey, T; United Kingdom, UK. North Africa- M, Morocco; Eurasia- I, Iran; Asia- China (S1, S2, S3, S7, S9, S11, S12, S13, S14 and S16); Japan, JP.

A total of 420 *P. parva* from 21 populations was sampled across *P. parva*’s native and invasive range (Table 1; Figure 1). *P. parva* kidney DNA was extracted to investigate the presence of *S. destruens* in the fish tissue as the parasite is found at a high prevalence in this organ.^21^ All *P. parva* samples were collected during 2009–2010 and were fixed in 92% ethanol upon collection. The DNA was extracted using Qiagen DNeasy Blood and Tissue kit following the manufacturer’s protocol. *S. destruens* 18S rRNA was amplified using the method described in Mendonca and Arkush,^22^ which is currently the most sensitive DNA-based detection method for this parasite. All samples identified as positive for *S. destruens* DNA using 18S rRNA were also sequenced for the ITS 1 and mitochondrial DNA Cyt-b regions (total length of 700 bp, spanning the Cyt-b gene (629 bp), the Cyt-b-COI intergenic region (60 bp) and the COI gene (11 bp)) in order to investigate the putative origin of *S. destruens*. Disease prevalence was calculated as: (number of *S. destruens* positive fish/total number of fish tested) × 100.

The nested PCR for the ITS 1 region (623 bp) used the primers Sdes2F (5′-CTT CGG ATT GGC CCT GTA C-3′)^14^ and NC 2 (5′-TTA GTT TCT TTT CCT CCG CT-3)^23^ in the first step-PCR, and Sdes2F and SD-ITS R1 (5′- CGA TGC ACG AGC CAA GAG-3′)^9^ in the second step-PCR. The PCR conditions were 1 × Promega (Madison, WI, USA) Flexi buffer, 1.5 mM MgCl_2_, 0.2 mM dNTPs, 0.3 μM primers and 0.5 U *Taq* polymerase (Promega). PCR cycling conditions included an initial denaturation at 95 °C for 3 min, followed by 35 cycles of 30 s at 95 °C, 45 s at X °C and 90 s at 62 °C, and final extension at 62 °C for 7 min (X=annealing temperatures were 60 °C and 59 °C for the first and the second PCR, respectively). The amplified product was gel extracted before sequencing. Cyt-b (700 bp) sequences were obtained for *S. destruens* using nested PCR. The primers used were Nt-Cytb-F1 (5-ATG AGT TTA TGG GGA GCG) coupled with Nt-Cytb-R1 (5-GCT CCA GCC AAC ACA GGT AAG GAT AAT AAC) in the first step-PCR. The second step-PCR used the primer Nt-Cytb-F2 (5-GGA GGG TTT AGT GTG GAT AAT GC) coupled with Nt-Cytb-R1 (5-TCA TCG TCA AAT CCA ACT CAC C). The PCR conditions were 1 × Promega Flexi buffer, 1.5 mM MgCl_2_, 0.2 mM dNTPs, 0.3 μM forward and reverse primer and 0.5 U *Taq* polymerase (Promega). Cycling conditions included an initial denaturation at 95 °C for 2 min, followed by 35 cycles with 95 °C for 40 s, X°C for 40 s, and 72 °C for 60 s, and a final extension step at 72 °C for 5 min (X=annealing temperatures were 56 °C and 58 °C for the first and the second PCR step, respectively).

Sequences were cleaned and aligned using Clustal W in BioEdit Version 5.0.9.^24^ A phylogenetic network was generated for the 18S rRNA marker using DnaSP version 5.10,^25^ and Network and Network Publisher^26^ (Available at: http://www.fluxus-engineeering.com) using the sequences generated in the present study and all the published sequences available for this marker (FN996945.1, AY267344.1, AY267345.1, and AY267346.1^13, 19^). A phylogenetic tree was drawn for the ITS 1 marker using MrBayes^27^ with the sequences generated in the present study and the sequences available in GenBank (FJ440707.1, FJ440708.1, FJ440709.1, FJ440702.1, FJ440703.1, FJ440704.1 and KT361608.1^9, 14^). The best model fitting our data was Hasegawa–Kishino–Yano (HKY), and it was investigated with jModel test v2.1.4.^28^ The number of haplotypes and haplotype diversity were calculated using DnaSP.^25^ All the sequences obtained through this work have been deposited in GenBank (MF138119, MF062546-MF062560 and MF101749-MF101755).

### *Pseudorasbora parva* Cyt-b sequencing

PCR amplification of the Cyt-b gene was carried out on *P. parva* individuals that had tested positive for *S. destruens* using the primers L15267 (5-AAT GAC TTG AAG AAC CAC CGT-3′) and H15891Ph (5-GTT TGA TCC CGT TTC GTG TA-3′)^29^ with an amplicon size of ~600 bp. The reaction conditions included a 50-μL reaction volume with 100 ng of template DNA, 1 × Promega Flexi buffer, 2 mM MgCl_2_, 0.2 mM dNTPs, 0.3 μM forward and reverse primer and 0.5 U *Taq* polymerase. The cycling conditions included an initial denaturation at 95 °C for 15 min followed by 35 cycles of 30 s at 95 °C, 90 s at 60 °C and 60 s at 72 °C, with a final elongation step at 72 °C for 15 min.

The Cyt-b sequences for *P. parva* that had tested positive for *S. destruens* were aligned with all available *P. parva* Cyt-b sequences across populations in its native range and invasive range^30^ (JF489575–JF489887, and KR074432–KR074994). These populations included the populations tested for *S. destruens* in this study. The sequences were aligned by Clustal W in BioEdit.^24^ Haplotype diversity was calculated in DnaSP version 5.10.^25^ A phylogenetic tree was constructed to identify the *P. parva* haplotypes associated with the presence of *S. destruens*. The phylogenetic analysis was performed using MrBayes,^27^ and posterior probabilities were obtained after 2 500 000 generations with a burn-in of 25%. The tree was calculated using a Hasegawa–Kishino–Yano model with a Gamma distribution (HKY+G) model^31^ determined with jModel test v 2.1.4.^28^ The Cyt-b gene sequences from *Ictiobus bubalus* (JF799443.1), *Hypentelium nigricans* (JF799441.1) and *Danio rerio* (JN234356.1) were used as outgroups.

### Population demographic analysis

The association between *S. destruens* and *P. parva* in China was further investigated by inferring their demographic history. A strong association between the two species would result in them sharing a similar demographic history. This analysis was achieved by determining the mismatch distribution using the 18S rRNA gene for *S. destruens* and the Cyt-b gene for *P. parva*. This analysis plots the distribution of nucleotide differences between each pair of sequences and compares it to the expected values for a model of population expansion. A unimodal distribution is indicative of a population expansion in the recent past whereas a bimodal/multimodal distribution indicates that a population is at demographic equilibrium.^32^ Demographic changes were analyzed by calculating Harpending’s raggedness index (*Hri*), which quantifies the smoothness of the observed mismatch distribution^33^ and the sum of squared deviations between the observed and expected mismatch for the nucleotide differences^34^ in Arlequin version 3.5.^35^

Three statistical methods were used to test for the population expansion of *P. parva*: Fu’s *F*_s_ test,^36^ Tajima’s *D* test^37^ and Ramos-Onsins & Rozas’ *R*_2_ test.^38^ The *R*_2_ test was performed by DnaSP,^25^ and Fu’s *Fs* and Tajima’s *D* tests were carried out in Arlequin version 3.5.^35^ Only Tajima’s *D* test and the *R*_2_ test were performed for *S. destruens* as Fu’s *F*_s_ test is not suitable for small sample sizes.^38^ For Tajima’s *D* and *F*_s_, *P*-values were calculated based on a coalescent simulation algorithm, and for the *R*_2_ test, the *P*-values were based on parametric boot strapping with coalescence simulations.

## RESULTS

### Prevalence

Of the 10 Chinese populations tested in this study, 9 were found to be positive for the presence of *S. destruens* (Table 1 and Figure 1). The prevalence of this parasite in the Chinese populations ranged from 0 to 10%. The overall prevalence of *S. destruens* across all Chinese populations was 6% (12/200). *S. destruens* was also found in two European populations: Spain and the United Kingdom (Figure 1 and Table 1), with a prevalence of 5% (1/20) in both populations. Overall, the prevalence in Europe was 1.4% (2/140). *S. destruens* was not detected in Morocco, Iran or Japan.

### Genetic diversity of the parasite *S. destruens*

**Figure 2:**
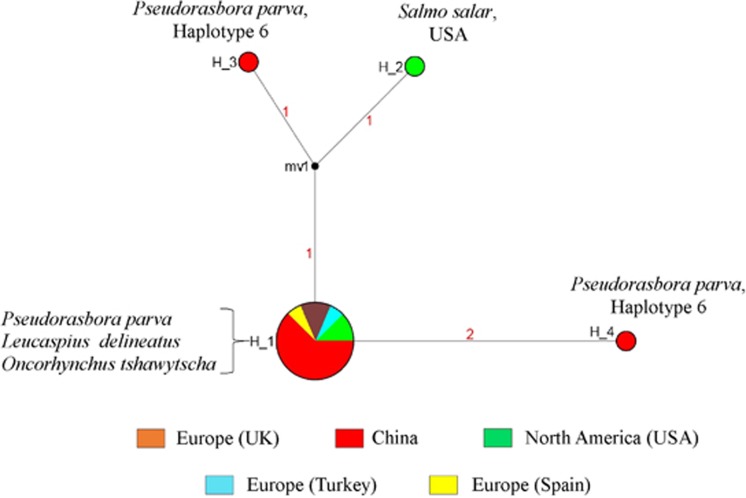
Minimum spanning network based on the 18S rRNA segment (397 bp) of *Sphaerothecum destruens*. The size of the different circles represents the frequency of each respective haplotype. The numbers on the branches indicate the number of mutations between the nodes. Black circles indicate branch splits. The color code indicates *S. destruens* individuals from different localities; Red: China (*n*=11), Yellow: Spain (*n*=1), Light blue: Turkey (*n*=1), Brown: UK (*n*=2) and Green: USA (*n*=3). Haplotypes H_1, H_2, H_3 and H_4 represent *S. destruens* 18S rRNA haplotypes. The Cyt-b *P. parva* haplotype for individuals positive for *S. destruens* (with *S. destruens* haplotypes H_3 and H_4) was Haplotype 6.

A sequence of 397 bp of 18S rRNA of *S. destruens* was obtained for 14 *P. parva* individuals from 11 different populations (Table 1), and it was aligned with the sequences published in GenBank (FN996945.1AY267344.1, AY267345.1 and AY267346.1^13, 19^). The haplotype diversity of *S. destruens* across all populations (native and invasive) was 0.22 with four identified haplotypes. Only three individuals, one from the USA (host: *S. salar*) and two from China (host: *P. parva*), were found to display different haplotypes (Figure 2).

**Figure 3:**
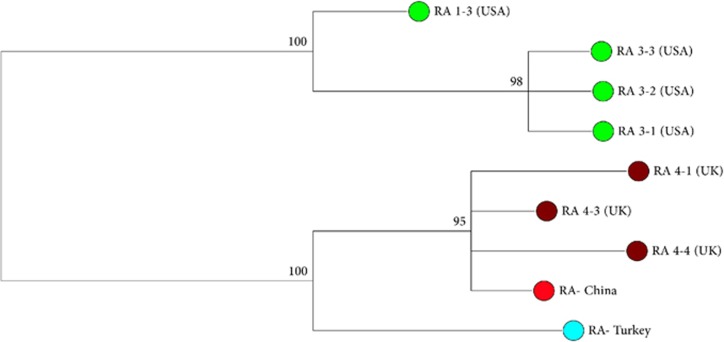
Unrooted phylogenetic tree resulting from Bayesian inference method in MrBayes^27^ based on Hasegawa–Kishino–Yano model^31^ analysis of the ribosomal ITS 1 sequences of *Sphaerothecum destruens*. Isolate origins and GenBank accession numbers are RA 1–3 (FJ440707.1), RA 3-1 (FJ440708.1), RA 3-2 (FJ440709.1), RA 3-3 (FJ440710.1), RA 4-1 (FJ440702.1), RA 4-3 (FJ440703.1), RA 4-4 (FJ440704.1), RA-Turkey (KT361608.1) and RA-China (MF138119).

We managed to sequence the ITS 1 region from only one of the 11 S*. destruens*-positive populations (Chinese population S3). However, when combined with the published ITS 1 sequences, the overall haplotypic diversity was high (Hd=0.97, Figure 3). Three clades were identified in our study. Individuals originating from the UK and China clustered together, the Turkish samples grouped by themselves, and the North American samples constituted the third clade (Figure 3). The Turkish samples were more closely related to the UK and China (S3) samples than the USA strains (Figure 3), indicating that the two European strains are closely related to the Chinese strain. In addition, a 700-bp fragment of *S. destruens* Cyt-b was successfully amplified in six *P. parva* populations (China (S1, S3, S11, S12 and S13) and the UK). No Cyt-b DNA sequence variation was found between the Chinese and the UK *S. destruens* samples (Supplementary Figure S1), which supported the IT S1 results. In combination, the close clustering between *S. destruens* isolated from Europe and China supports the hypothesis that the parasite was introduced to Europe from China.

### Genetic diversity of the host *P. parva*

**Figure 4:**
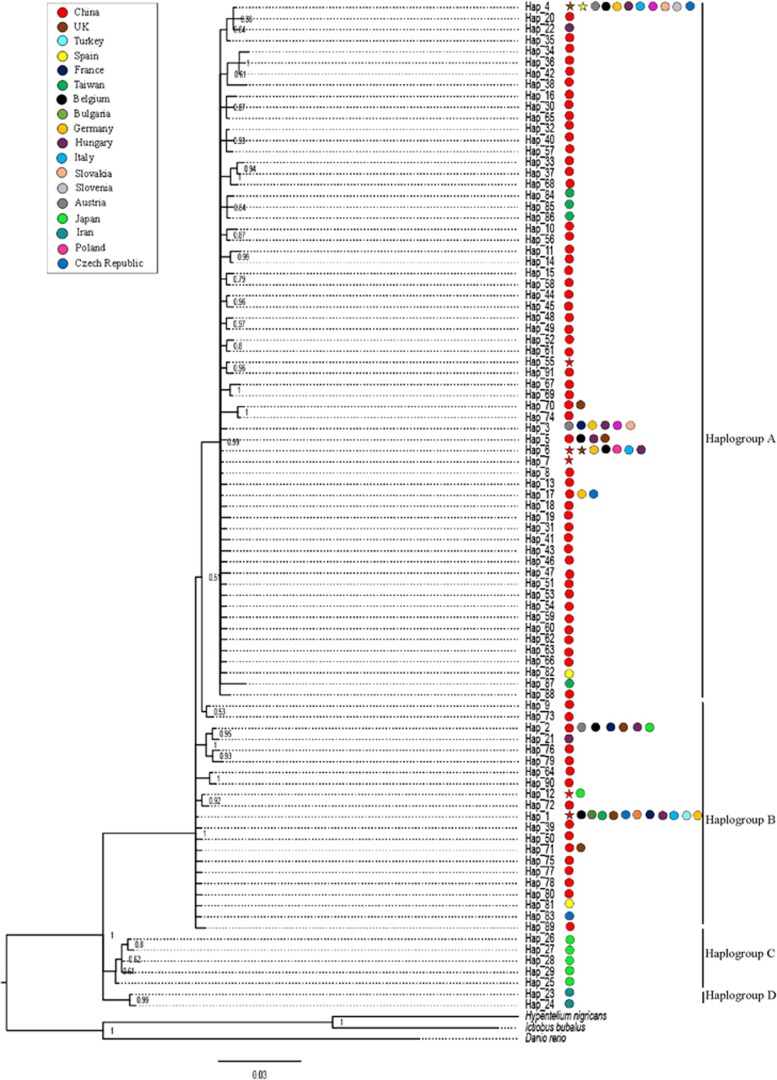
Molecular phylogenetic analysis of Cyt-b haplotypes of *Pseudorasbora parva* populations across its invasive and native range. The tree was inferred from the Bayesian inference method based on the Hasegawa–Kishino–Yano model^31^ with Gamma distribution in MrBayes.^27^ The colored circles indicate the countries that each haplotype has been found in and the colored stars indicate *S. destruens* positive haplotypes in that country.

A total of 91 haplotypes from 949 *P. parva* individuals were identified in the dataset of Cyt-b sequences of *P. parva* populations (a total of 62 *P. parva* populations) across its invasive and native range. The two main haplogroups identified in China were Haplogroups A and B (Figure 4). Six Cyt-b haplotypes in *P. parva* were found to be associated with the presence of *S. destruens* (Hap_1, Hap_ 4, Hap_6, Hap_7, Hap_12 and Hap_55; Figure 4). The highest number of *P. parva* individuals (*n*=7) that were positive for *S. destruens* had the Cyt-b haplotype Hap_6. The remaining haplotypes each had one *P. parva* individual positive for *S. destruens*.

### Demographic analysis of *P. parva* and *S. destruens*

**Figure 5:**
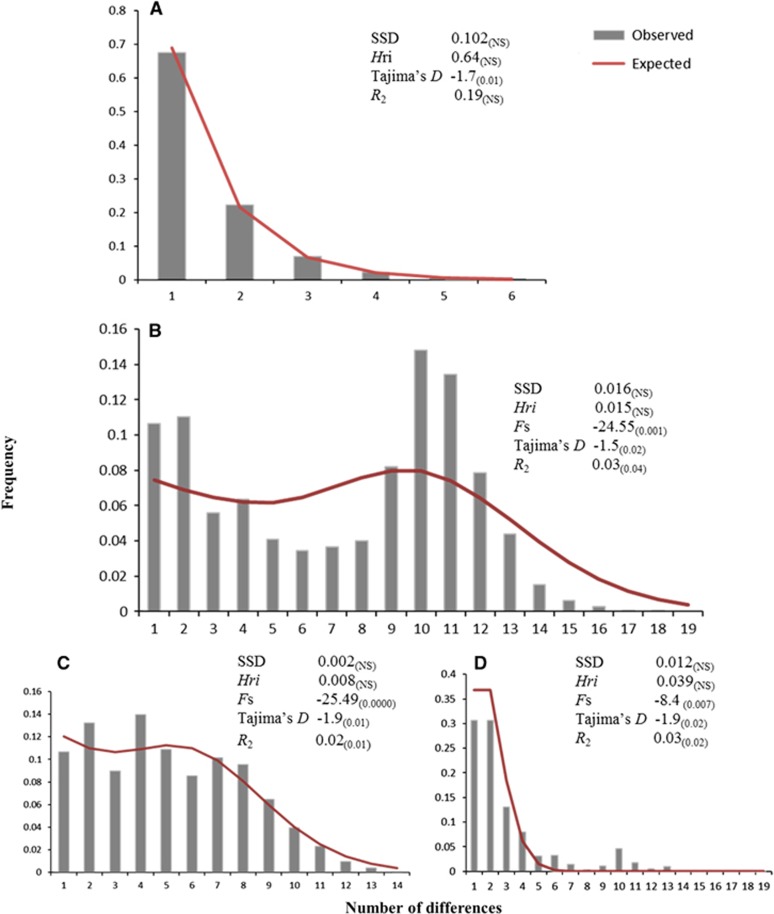
Frequency distributions of the number of pairwise nucleotide differences (mismatch) between (**A**) 18S rRNA haplotypes of *Sphaerothecum destruens* populations in China; (**B**) cytochrome b haplotypes of *Pseudorasbora parva* populations in China; (**C**) *P. parva* haplogroup A, and (**D**) *P. parva* haplogroup B. The solid line is the theoretical distribution under the hypothesis of population expansion. Sum of squared differences, Harpending’s raggedness index (*Hri*), Fu’s *F*_s_, Tajima’s *D* and Ramos-Onsins and Rozas’ *R*_2_ statistics are listed next to each dataset. The *P*-values for each statistical test can be found in parentheses. Significance was set at a *P*-value of 0.05, except for *F*_s_, for which it was set at 0.01.

The demographic analysis of the host, *P. parva*, and the parasite, *S. destruens*, suggested a potential recent population expansion in both species, supporting the close relationship between the two species. The sum of squared differences and Harpending’s raggedness index (*Hri*) were not significant for the pathogen and its host, indicating that the data are a relatively good fit with population expansion (Figure 5). Both species also had significant negative values for the Tajima’s *D* test, further supporting population expansion. *P. parva’s* population expansion was further supported by the *R*_2_ test. The *R*_2_ test, however, was not significant for the *S. destruens* population, which was in contrast to the negative value of Tajima’s *D* and the unimodal mismatch distribution.

The observed mismatch distribution for *P. parva* when all the populations are considered (Figure 5B) is bimodal. The bimodality of the mismatch distribution for *P. parva* could be due to the presence of different haplogroups.^39^ In China, there are two main established haplogroups, A and B (Figure 4), despite the statistical test supporting that the population as a whole has undergone a recent population expansion. When the data were split by haplogroups, the mismatch distribution was unimodal (Figures 5C and 5D).

## DISCUSSION

Our results support that *S. destruens* was introduced to Europe from China via its reservoir host *P. parva*, designating *S. destruens* as a non-native parasite to Europe. As a non-native parasite to Europe, *S. destruens* will need to be risk assessed^40^ with respective fish movement limitations on fishes that test positive for the parasite. This is especially important in regions where *S. destruens* has been associated with population declines of native freshwater fishes.^9, 10^ The healthy reservoir of *S. destruens, P. parva*, has been accidentally introduced to Europe through the aquaculture trade of Asian carp (*Hypophthalmichthys molitri*, *Ctenopharyngodonidella*).^11^ It is often challenging to determine whether a parasite was introduced, as parasitological surveys are neither regular nor exhaustive.^41^ The disseminated form of disease caused by *S. destruens* is difficult to detect through traditional histo-parasitological surveys, which focus on gross pathological changes in the organs. Thus, we used molecular tools to test the hypothesis that *S. destruens* was introduced to Europe using an extensive survey of *P. parva* across its native and invasive range.

No geographical isolation was identified between the Chinese and the European *S. destruens* populations using the phylogenetically informative marker ITS 1. The results indicate that there are two geographical clades for *S. destruens,* one encompassing the samples from North America and one including the samples from China, the UK and Turkey. However, within the European clade, the UK and China sequences were more closely related compared with the sequence from Turkey. This similarity can be explained by the invasion history of the parasite’s reservoir host, *P. parva*. The UK populations of *P. parva* genetically group with *P. parva* populations found north of the River Yangtze (Haplogroup A; Figure 4). However, the *P. parva* found in Turkey is genetically more similar to populations found to the south of the River Yangtze^42^ (Haplogroup B; Figure 4), which could explain why the Turkish *S. destruens* isolate (Figure 3) grouped on its own within the overall European and China clade. Thus, the genetic diversity of the host species, *P. parva*, is likely to reflect the observed diversity of *S. destruens*, further supporting the hypothesis that the *S. destruens* populations found in Europe have been introduced from China.

Using two nuclear and one mitochondrial marker, we demonstrated that *S. destruens* is widely distributed in China (present in 90% of the sampled Chinese *P. parva* populations). A higher presence of the parasite in its native range would be expected because the process of species introduction can lead to the loss of associated parasites through stochastic effects.^43, 44^ The wide distribution of the *P. parva* populations that are positive for *S. destruens* across China suggests that the two species could share a long co-evolutionary history. Similarly, the emergence and association of fungal parasites with reservoir hosts over a long evolutionary history has been recently demonstrated for the chytrid fungus *Batrachochytrium salamandrivorans.*^45^ *B. salamandrivorans* has likely originated and coexisted with its reservoir hosts for millions of years in Asia before being introduced across the world with the trade of its reservoir hosts.^45^ Similar to *B. salamandrivorans*, *S. destruens* appears to have been introduced to Europe via the accidental introduction of its reservoir host.

The demographic analysis of *P. parva* and *S. destruens* partially suggests that both species have undergone a recent population expansion. The partial congruence between the demographic history of the two species is surprising, especially in light of the true generalist nature of *S. destruens* and its ability to use a number of different hosts. Recent work, however, has indicated that, following the establishment of a generalist parasite in a community, its population dynamics are driven via intra-host transmission rather than by inter-host transmission.^46, 47^ This could explain the observed similarity in the population demographic history of the two species.

This study represents the first screening of the native and invasive *P. parva* populations for the presence of *S. destruens*. It is important to note that the prevalence values recorded in positive populations are very likely to be underestimates of the true prevalence of this parasite, as only the kidney was sampled. *S. destruens* infects multiple organs and does so unequally.^21^ This lack of infection localization makes this organism harder to detect. Populations that have been detected as negative for *S. destruens* in this study must be treated with caution as it cannot be excluded that the parasite might still be present in these and other populations in the country. For example, the Turkish population sampled in this study was found to be negative for *S. destruens*, but the parasite has been detected in another *P. parva* population in Turkey.^9^ The screening recommendations for *S. destruens* include sampling multiple populations, and where possible, a minimum sample size of 30 fish should be tested and multiple tissues (kidney, liver, testis/ova) should be tested to increase the probability of detection.^40^

Currently, there are 14 known species that are susceptible to *S. destruens*, including valuable aquaculture species (salmon, carp and sea bass)^9, 12, 13^ and endemic fishes to Europe that are of high conservation value (*L. delineatus*, *Oxynoemacheilus* sp., *Petroleuciscus smyrnaeus*, *S. fellowesii* and *Dicentrarchus labrax*).^9, 21^ The close association of the reservoir host, *P. parva*, with aquaculture facilities (due to its accidental introduction along with carp from China)^11^ and the ability of the parasite to establish in local freshwater communities within a year of its introduction^46^ increases the risk of disease to native fishes. In the last 20 years, aquaculture production has increased exponentially to support economic growth, with its expansion being highly reliant on non-native species.^48^ The introduction of non-native species can be detrimental both to ecosystem services and naive communities.^49^ The potential threats associated with aquaculture production and the resultant fish movements highlight the importance of risk assessments to identify emergent parasites. Horizon scanning for potential emergent diseases will be critical in informing strict biosecurity controls in order to prevent disease introduction.

## Figures and Tables

**Table 1:** Sampled populations of *Pseudorasbora parva* and the distribution of *Sphaerothecum destruens* in *P. parva* using molecular detection across *P. parva*’s native and non-native range

**Population**	**Code**	**Coordinates**		**Prevalence of** ***S. destruens***	**Successfully amplified markers for every positive sample**		
		**X**	**Y**		**18S rRNA**	**Cyt-b**	**ITS 1**
China 1	S1	115.56	37.55	10% (2/20)	✓	✓	
					✓		
China 2	S2	117.12	34.81	5% (1/20)	✓		
China 3	S3	118.59	33.19	5% (1/20)	✓	✓	✓
China 7	S7	110.32	25.27	0% (0/20)			
China 9	S9	113.11	29.15	5% (1/20)	✓		
China 11	S11	110.99	34.62	5% (1/20)	✓	✓	
					✓	✓	
China 12	S12	117	38.7	10% (2/20)	✓	✓	
					✓		
China 13	S13	122.52	40.1	5% (1/20)	✓	✓	
					✓	✓	
China 14	S14	124.99	45.03	10% (2/20)	✓		
					✓		
China 16	S16	118.27	40.9	5% (1/20)	✓		
Austria	A	14.72	48.19	0% (0/20)			
Bulgaria	BG	43	26	0% (0/20)			
France	F	−1.73	47.1	0% (0/20)			
Iran	IR	54.78	37.05	0% (0/20)			
Italy	IT	10	44	0% (0/20)			
Japan	JP	139.43	35.67	0% (0/20)			
Morocco	M	32.11	2.89	0% (0/20)			
Spain	SE	0.86	40.7	5% (1/20)	✓		
Turkey	T	30.04	40.91	0% (0/20)			
United Kingdom	UK	1	51	5% (1/20)	✓	✓	
Hungary	H	18	46	0% (0/20)			
