# Supplementary figures and images for: Origin and invasion of the emerging infectious pathogen Sphaerothecum destruens
Source: Emerg Microbes Infect. 2017 Aug 23;6(8):e76–. doi: 10.1038/emi.2017.64 (PMC5583672; doi:10.1038/emi.2017.64)

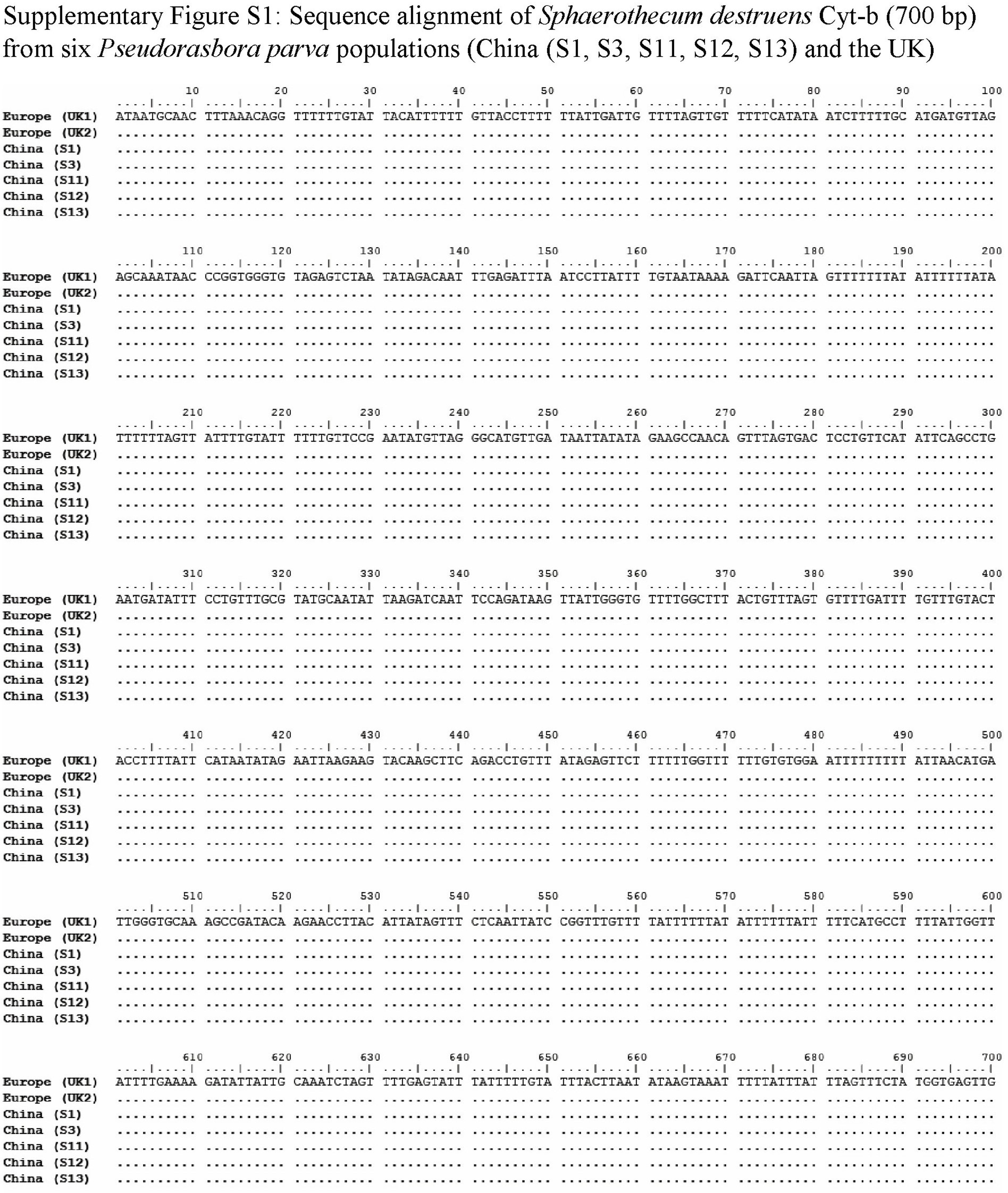

Supplement: Supplementary Figure S1 [file emi201764x1.tif]
